# VSSP-activated macrophages mediate senescence and tumor inhibition in a preclinical model of advanced prostate cancer

**DOI:** 10.1186/s12964-023-01095-3

**Published:** 2023-04-13

**Authors:** Rydell Alvarez-Arzola, Nicoló Bancaro, Ping Lai, Giuseppe Attanasio, Laura Pellegrini, Martina Troiani, Manuel Colucci, Simone Mosole, Emiliano Pasquini, Andrea Alimonti, Circe Mesa

**Affiliations:** 1 Department of Immunoregulation, Immunology and Immunotherapy Direction, Center of Molecular Immunology, Havana, Cuba; 2 Department of Molecular Oncology, Institute of Oncology Research (IOR), 6500 Bellinzona, Switzerland; 3 Faculty of Medicine, Università della Svizzera Italiana, 1011 Lugano, Switzerland; 4 Department of Medicine, University of Padua, 35131 Padua, Italy; 5 Medical Oncology, Oncology Institute of Southern Switzerland, 6500 Bellinzona, Switzerland; 6 Innovative Immunotherapy Alliance S.A., Mariel, Artemisa Cuba

**Keywords:** Macrophages, VSSP, Prostate cancer, Adoptive transfer

## Abstract

Androgen deprivation therapy (ADT) is a standard therapy for prostate cancer (PCa). Though disseminated disease is initially sensitive to ADT, an important fraction of the patients progresses to castration-resistant prostate cancer (CRPC). For this reason, the identification of novel effective therapies for treating CRPC is needed. Immunotherapeutic strategies focused on macrophages as antitumor effectors, directly enhancing their tumoricidal potential at the tumor microenvironment or their adoptive transfer after ex vivo activation, have arisen as promising therapies in several cancer types. Despite several approaches centered on the activation of tumor-associated macrophages (TAMs) in PCa are under investigation, to date there is no evidence of clinical benefit in patients. In addition, the evidence of the effectiveness of macrophage adoptive transfer on PCa is poor. Here we find that VSSP, an immunomodulator of the myeloid system, decreases TAMs and inhibits prostatic tumor growth when administered to castrated Pten-deficient prostate tumor-bearing mice. In mice bearing castration-resistant Pten^pc−/−^; Trp53^pc−/−^ tumors, VSSP administration showed no effect. Nevertheless, adoptive transfer of macrophages activated ex vivo with VSSP inhibited Pten^pc−/−^; Trp53^pc−/−^ tumor growth through reduction of angiogenesis and tumor cell proliferation and induction of senescence. Taken together, our results highlight the rationale of exploiting macrophage functional programming as a promising strategy for CRPC therapy, with particular emphasis on ex vivo-activated proinflammatory macrophage adoptive transfer.

**Graphical abstract:**

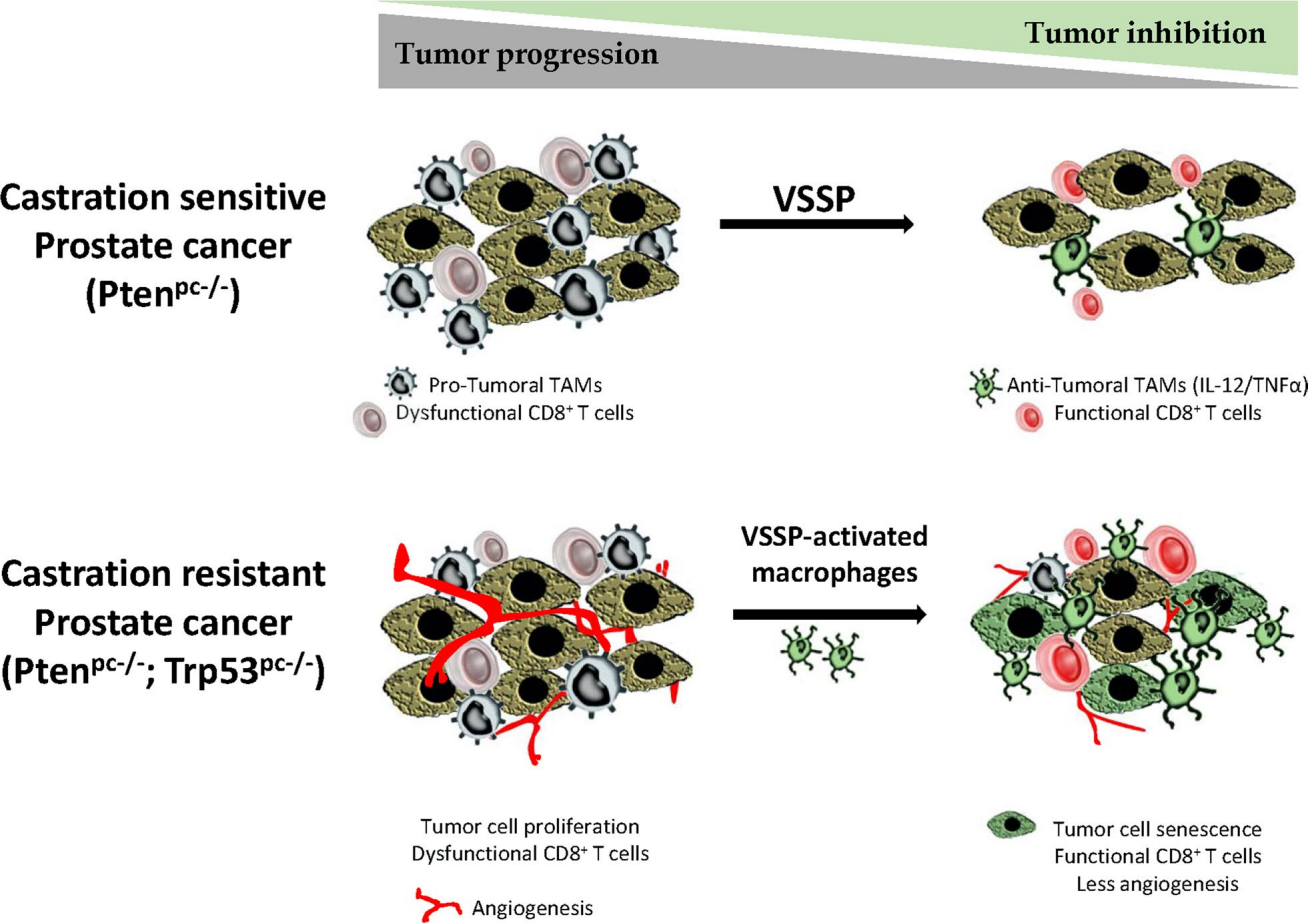

**Video abstract**

**Supplementary Information:**

The online version contains supplementary material available at 10.1186/s12964-023-01095-3.

## Introduction

Prostate cancer (PCa) is the most common non-cutaneous cancer type and the second leading cause of male cancer mortality [1]. In USA, the survival rate at 5 years of prostate cancer (PCa) patients with distant metastasis is 29.8% [2]. Androgen deprivation therapy (ADT) is a standard therapy for PCa at different stages of the disease, however, thought disseminated disease is initially sensitive to ADT, an important fraction of the patients progresses and develop castration-resistant prostate cancer (CRPC) [3, 4]. In this setting, the most advanced treatments provide less than 3 years of median survival with a significant detriment of life quality due to secondary effects related to treatments [5]. For these reasons, the identification of novel effective therapies for treating CRPC is needed.

Immunotherapy (IT) based on reactivation of T cells in the tumor microenvironment (TME) is emerging as an effective option to treat cancer. However, due to the low genetic mutational load, the presence of specific genetic alterations that influence the immune landscape, and the scarce presence of tumor-infiltrating lymphocytes, prostate tumors are considered poorly responsive to IT [6, 7]. Contrary to what occurs with T cells, tumor-associated macrophages (TAMs) are the most abundant noncancerous cell type in the TME [3]. Actually, tumor-associated macrophages (TAMs) and other cells of myeloid origin represent up to 70% of the infiltrating immune cells in the prostate cancer microenvironment [8]. Macrophages are functionally plastic immune cells that can be classically activated to inflammatory cells with antitumor activity (M1) or alternatively activated to anti-inflammatory and pro-tumorigenic cells (M2) [9–12]. Because of the complexity of signals derived from the TME and the macrophage plasticity, TAMs can exhibit a mixed and heterogeneous phenotype, usually M2-like. However, the functional plasticity of macrophages can be exploited to induce their functional reprogramming towards antitumor effectors in the TME [3]. In addition, novel macrophage-based strategies are under investigation, including adoptive transfer of ex vivo stimulated macrophages [4, 13]. Despite several preclinical studies suggesting that functional reprogramming of TAMs in PCa could be an effective anticancer strategy, the amount of evidence is still limited, while there is a lackof evidence regarding the efficacy of macrophage adoptive transfer in this scenario.

Very small size particles (VSSP) are a nanoparticulated drug composed of outer membrane vesicles of gram-negative bacteria *Neisseria meningitidis* and the GM3 ganglioside [14]. In mice, VSSP induces myelopoiesis with a significant differentiation shift to the monocytic-macrophage lineage after chemotherapy-induced myeloablation; matures and activates dendritic cells; abrogates the suppressive activity of myeloid-derived suppressor cells, and induces their differentiation to stimulatory antigen-presenting cells in several scenarios including tumor-bearing mice [15–21]. Recent studies of our group also demonstrate that VSSP polarizes macrophages to an M1 phenotype with tumoricidal activity in breast tumor-bearing mice (Alvarez-Arzola et al. manuscript in revision). Based on these immunomodulatory properties, we hypothesized that VSSP could activate naïve macrophages in vitro and re-educate TAMs in vivo, to potentiate their tumor-suppressive activity, therefore inhibiting the proliferation of aggressive prostate cancer.

The present study aimed to determine the effect of immunotherapies based on VSSP-educated macrophages, using VSSP as immunomodulatory immunotherapy or ACT of macrophages activated ex vivo with VSSP, in prostate tumor-bearing mice.

## Materials and methods

### Mice

All mice were maintained under specific pathogen-free conditions at the IRB institute and the experiments were performed according to state guidelines and approved by the local ethical committee (“Dipartimento della Sanità e Socialità, Esperimenti su animali,” Canton Ticino). Pten^pc−/−^ and Pten^pc−/−^; Trp53^pc−/−^ mice (C57BL/6 J background) were generated and genotyped for Cre as previously described [3, 8]. Female Pten^loxP/loxP^ and female Pten^loxP/loxP^; Trp53^loxP/loxP^ mice were crossed with male PB-Cre4 transgenic mice and genotyped for Cre using following primers: primer 1 (5′-AAAAGTTCCCCTGCTGATGATTTG T-3′) and primer 2 (5′-TGTTTTTGACCAATTAAAGTAGGCTGTG-3′) for Pten^loxP/loxP^; primer 1 (5′-TGATGGACATGTTCAGGGATC-3′) and primer 2 (5′-CAGCCACCAGCTTGCATGA-3′) for Probasin-CRE. Surgical castration was performed under anesthesia with isoflurane. Male Pten^pc−/−^ mice were 9–12 weeks old at the time of castration. Mice were monitored for recovery from anesthesia and checked daily for 4 days postoperatively. Surgical skin clips were removed on postoperative day 5. Mice undergoing treatment were administered control vehicle or therapeutic doses of the appropriate agents. Any mouse suffering distress or greater than 15% weight loss during treatment was euthanized by CO_2_ asphyxiation. At the completion of the study, mice were euthanized by CO_2_ asphyxiation and tissues were collected for histology analysis and single-cell suspensions for flow cytometry. The volume and area of the tumor were estimated at completion of the study using the formulas:

$$\begin{aligned} Volume & = R1*R2*R3*4/3\pi \\ Area & = R1*R2*\pi \\ \end{aligned}$$

where R1 and R2 are the longitudinal and lateral radii and R3 is the thickness of the tumor. We also measured the tumor weight in the experiments using Pten^pc−/−^; Trp53^pc−/−^ mice.

### Treatments

VSSP was produced at the center of molecular immunology (CIM) with procedures reported elsewhere [14]. For the in vivo experiments using Pten^pc−/−^ and Pten^pc−/−^; Trp53^pc−/−^ mice, VSSP was administered two times per week (on Mondays and Thursdays) by intraperitoneal injections at a final concentration of 5 mg/kg. αCXCR2 (AZD5069; Astrazeneca) was administered daily by intraperitoneal injections at a final concentration of 100 mg/kg from Monday to Friday only to Pten^pc−/−^. Control animals received vehicle (PBS). For the macrophage adoptive transfer experiments, 2–5 × 10^6^ VSSP-activated bone marrow-derived macrophages (BMDMs) were intravenously administered to Pten^pc−/−^; Trp53^pc−/−^ mice once per week for 12 weeks. Control mice received the same amounts of unstimulated BMDMs.

### Cell lines

The Trp53-inactivated prostate epithelial cells TRAMP-C1 cell line was purchased from ATCC. TRAMP-C1 Pten-null variant (TRAMP-C1-shPTEN) cell line was generated in the lab with short hairpin methodology and authenticated by WB and FACS for the deletion of Pten. Pten^pc−/−^; Trp53^pc−/−^ primary cancer cells were generated in the lab. Freshly collected prostate tumor tissue was placed in a sterile 60 mm dish and gently washed several times with HBSS to remove any contaminant from the collection process. The tissue was then mechanically disrupted with crossed scalpels, transferred to a 50 mL tube, and incubated in 5 mL of a digestion medium at 37 °C for 2 h on a rotator (140 rpm). The digestion medium contained Dulbecco modified Eagle’s medium (DMEM)/F12 (Gibco), 10% fetal bovine serum (Gibco), 1 mg/mL collagenase D (Sigma-Aldrich), 1 mg/mL hyaluronidase (Sigma-Aldrich), and 1 μg/mL DNase I (Sigma-Aldrich). Then, the digestion mixture was washed by centrifugation, and the pellet obtained was resuspended in HBSS medium. This process was repeated three times. The cell suspension was then plated in a 10 cm dishes and cultured in DMEM high glucose w/l-glutamine media (Gibco) supplemented with 5% fetal bovine serum, 5 µg/mL insulin (Sigma-Aldrich) and 6 ng/mL recombinant human epidermal growth factor (rhEGF) (Promega). During early passage, the cells were differentially trypsinized to enrich for more adherent epithelial cells. All the above-mentioned cell lines were maintained at 5% CO_2_ at 37 °C and cultured in DMEM with 10% heat-inactivated FBS, and regularly tested for mycoplasma (MycoAlert Mycoplasma Detection kit).

### In vitro differentiation of BMDMs

Bone marrow cells were collected by flushing femurs and tibias. Red blood cells were lysed using ACK lysis buffer (Gibco). Cells were cultured with RPMI (Gibco) with 10% heat-inactivated FBS containing M-CSF (30 ng/mL) (PeproTech) for 5 days. BMDMs were obtained after the removal of the floating cells and washing the culture plates with PBS. For in vitro stimulation, at day 5 cells were washed, and media was replaced by RPMI containing 10 µg/mL of VSSP. Cells were cultured for 24 h. Next, cells were either used for FACS, cultured for 24 h with RPMI with 10% heat-inactivated FBS to collect the conditioned media (CM) or used for the adoptive transfer. IL-4, IL-13 and CXCL2 were obtained from PeproTech.

### In vitro culture experiments

Prostate cancer cell lines were cultured with RPMI 1640 containing 10% heat-inactivated FBS (normal medium), conditioned medium from BMDMs (1 part of normal medium and 1 part of unstimulated BMDMs conditioned medium) or conditioned medium from VSSP-activated BMDMs (1 part of normal medium and 1 part of VSSP-activated BMDMs CM). Then the cells were analyzed for Crystal Violet assay and/or for β-Galactosidase activity assay (after 72 h of culture, fold change compared with normal medium) (Cell Signaling Technology).

### Immune tumor microenvironment characterization

Tumors were disaggregated and digested in collagenase D and DNAse for 30 min at 37 °C to obtain single-cell suspension. For intracellular cytokine detection, cells where stimulated for 5 h with PMA/ionomycin plus Golgi Plug (BD). After neutralization of unspecific binding with αCD16/CD32 (clone 93, Biolegend), single-cell suspensions were stained with directly conjugated specific monoclonal antibodies to assess the phenotype. The antibodies used were: αCD45 BV605 (clone 30-F11); αLy-6G FITC (clone 1A8); αLy6C PE-Cy7 (clone HK1.4), αCD11b BV421 (clone M1/70); αF4/80 PE (clone BM8), αCD11c BV650 (clone N418), αCD19 APC-Cy7 (clone 6D5), αCD3 BV421 (clone 145-2C11), αCD8 BV650 (clone 53-6.7), αCD4 PerCP-Cy5.5 (clone GK1.5), αNK1.1 PE (clone PK136), αCD11c BV650 (N418), αCD80 FITC (clone 2D10), αTNFα BV785 (clone MP6-XT22) and αIL-12 p40 PE (clone C17.8). For flow gating we used isotype controls and florescence minus one controls. All the antibodies were purchased from eBioscience or Biolegend. Samples were acquired on a BD Fortessa flow cytometer (BD Biosciences). Data were analyzed using FlowJo software (TreeStar Inc.).

### Immunohistochemistry

For immunohistochemistry (IHC), tissues were fixed in 10% formalin (ThermoScientific) and embedded in paraffin following standard procedures. Sections of 4 μm were used for IHC analyses and hematoxylin and eosin (H&E) staining. Once dried, the sections were treated with OTTIX plus solution and OTTIX shaper solution to dewax and rehydrate the sections. All the above-mentioned reagents were obtained from Diapath. Antigen retrieval was performed using pH 6 solutions at 98 °C for 20 min. Next, the endogenous peroxidases and non-specific-binding sites were blocked using 3% H_2_O_2_ (VWR chemicals) and Protein-Block solution (DAKO Agilent technologies) respectively, for 10 min. Sections were then stained for anti-p16 (ab211542, Abcam, 1:1200), anti-H2AX (CST #7631S, 1:200), anti-PAI-1 (CST #11907S, 1:200), anti-F4/80 (CST #30325S, 1:200), anti-PHP1γ (CST #2600S, 1:200), anti-Ki67 (Clone SP6; Lab Vision Corporation #RT-9106-R7, RTU, 1:200), anti-CD31 (CST #3528S, 1:200) and anti-CC3 (CST #9661S, 1:200). IHC analyses were performed using the Imagescope software.

### Statistical analysis

Data were analyzed using GraphPad Prism (version 7). Data were recorded as the mean ± standard deviation (SD) or standard error of the mean (SEM) of the indicated biologic or experimental replicates as indicated in the figure legends. All experiments were repeated at least two times with similar results, and representative experiments are shown in the figures. Statistical differences were analyzed by Student’s t test (two groups) or by one-way ANOVA and Tukey test (more than two groups) for pairwise comparisons. Differences were considered statistically significant at *p* < 0.05.

## Results

### In vivo administration of VSSP reduces TAMs and inhibits the growth of Pten-deficient Trp53 wild type prostate tumors

To investigate the antitumor effect of VSSP on PCa, we used two Pten-null prostate conditional (pc^−/−^) mouse models, in which Trp53 expression in the prostate is respectively maintained or depleted [22, 23]. Both models recapitulate pathological and molecular features of human prostate cancer at different stages of progression, with Pten^pc−/−^; Trp53^pc−/−^ tumors more aggressive compared to Pten^pc−/−^; Trp53^pc+/+^ (Pten^pc−/−^). Pten^pc−/−^; Trp53^pc−/−^ tumors are resistant to ADT, while surgical castration in Pten^pc−/−^ mice promotes initial tumor regression followed by tumor progression and the emergence of castration-resistant prostate tumors [3, 8].

**Fig. 1:**
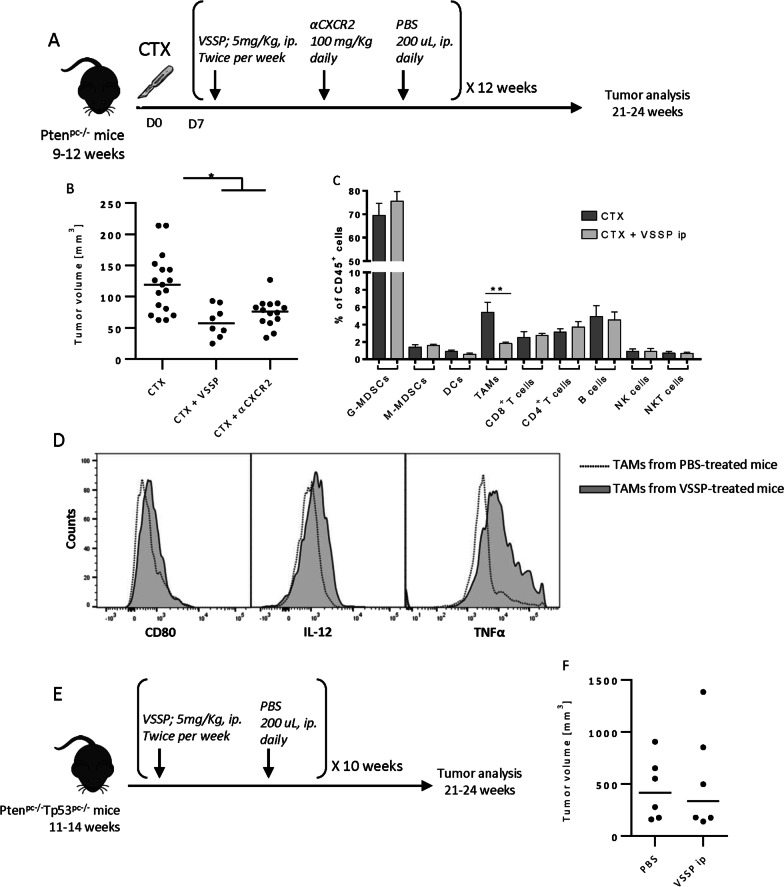
In vivo administration of VSSP reduces prostate tumor growth in Pten^pc−/−^. **a** Schedule of treatment used in the pre-clinical trial in Pten^pc−/−^ mice. 9–12 weeks old mice were surgically castrated and 7 days after started the administration of VSSP (5 mg/kg, i.p, twice per week), αCXCR2 (100 mg/kg, orally, daily) or vehicle (PBS, 200 µL; i.p. or orally). After 12 weeks of treatment mice were euthanized and tumors were collected. **b** Prostate tumor volume of Pten^pc−/−^ mice treated with VSSP (n = 8), αCXCR2 (n = 14) or PBS (n = 17) after surgical castration. **c** FACS quantitation of TAMs (CD45^+^CD11b^+^Ly6G^−^F4/80^+^). **d** Phenotype of TAMs determined by FACS. **e** Schedule of treatment used in the pre-clinical trial with Pten^pc−/−^; Trp53^pc−/−^ mice. 11–14 weeks old Pten^pc−/−^; Trp53^pc−/−^ mice were treated with VSSP (5 mg/kg, i.p, twice per week) or PBS (200µL; i.p., twice per week). After 10 weeks of treatment mice were euthanized and tumors were collected. **f** Prostate tumor volume of Pten^pc−/−^; Trp53^pc−/−^ mice treated with VSSP (n = 6) or PBS (n = 6). Symbols indicates significant differences by Tukey test (*p < 0.05; **p < 0.01)*.* *CTX* surgical castration, *TAMs* tumor-associated macrophages

To evaluate the in vivo effect of VSSP, we started with the less aggressive Pten^pc−/−^ model. Pten^pc−/−^ mice were surgically castrated, and 1 week after, we initiated the VSSP, αCXCR2 (AZD5069; selective CXCR2 antagonist under clinical evaluation [24]) or vehicle administration as depicted in Fig. 1a. Mice were sacrificed after 12 weeks of treatment. VSSP administration following castration significantly reduced tumor volume (Fig. 1b) and tumor weight (Additional file 1: Fig. S1a), a result comparable to CXCL2 inhibition (αCXCR2). No change in the body weight was observed in the Pten^pc−/−^ mice receiving VSSP (Additional file 1: Fig. S1b). To further determine if the VSSP affected TAMs content and/or frequency, we performed multiparametric flow cytometry on the tumors. The gating strategy for the immunoprofiling is shown in Additional file 1: Fig. S1c. The frequency of CD45 + CD11b + LY6G-F4/80 + TAMs were decreased in the VSSP-treated group compared to the untreated counterparts, while the remaining immune cell populations remained unaffected (Fig. 1c and Additional file 1: Fig. S1d). Additionally, the remaining macrophages detected in the tumor from the VSSP-treated mice exhibited an M1 phenotype characterized by an increased expression of CD80, IL-12 and TNFα (Fig. 1d). We also analyzed the same markers on G-MDSCs (Additional file 1: Fig. S1e) and M-MDSCs and DCs (data not shown) and we did not find any difference upon VSSP treatment. In order to determine if VSSP administration showed an effect on tumor-infiltrating lymphocytes, we determined by FACS the production of INFγ and Granzyme B (GraB) in the CD8+ subset. As shown in Additional file 1: Fig. S1f, CD8+ TILs produce higher amounts of INFγ and GraB in the VSSP-treated mice. We also evaluated the effect of VSSP administration in the immune system of the peritoneum, spleen and bone marrow and no significant changes were observed (data not shown).

Next, we decided to evaluate the effect of VSSP on castration-resistant Pten^pc−/−^; Trp53^pc−/−^ model, which, in addition to the increased aggressiveness, exhibits increased TAMs content compared to Pten^pc−/−^ tumors [3]. Tumor-bearing mice were treated as shown in Fig. 1e and sacrificed after 10 weeks of treatment. In this advanced scenario VSSP did not inhibit prostate tumor growth (Fig. 1f) and did not affected the TAMs frequency in the TME (data not shown). No change in the body weight was observed in the Pten^pc−/−^; Trp53^pc−/−^ mice receiving VSSP (Additional file 1: Fig. S1g). VSSP did not change either the frequency of immune cells in the peritoneum and the spleen of Pten^pc−/−^; Trp53^pc−/−^ tumor-bearing mice (data not shown). In summary, VSSP diminished the levels of TAMs and controlled tumor growth when administered to Pten^pc−/−^ tumor-bearing mice but was ineffective when administered to castration-resistant Pten^pc−/−^; Trp53^pc−/−^ tumor-bearing mice.

### Adoptive transfer of VSSP-stimulated BMDMs controls Pten^pc−/−^; Trp53^pc−/−^prostate tumor growth and aggressiveness

**Fig. 2:**
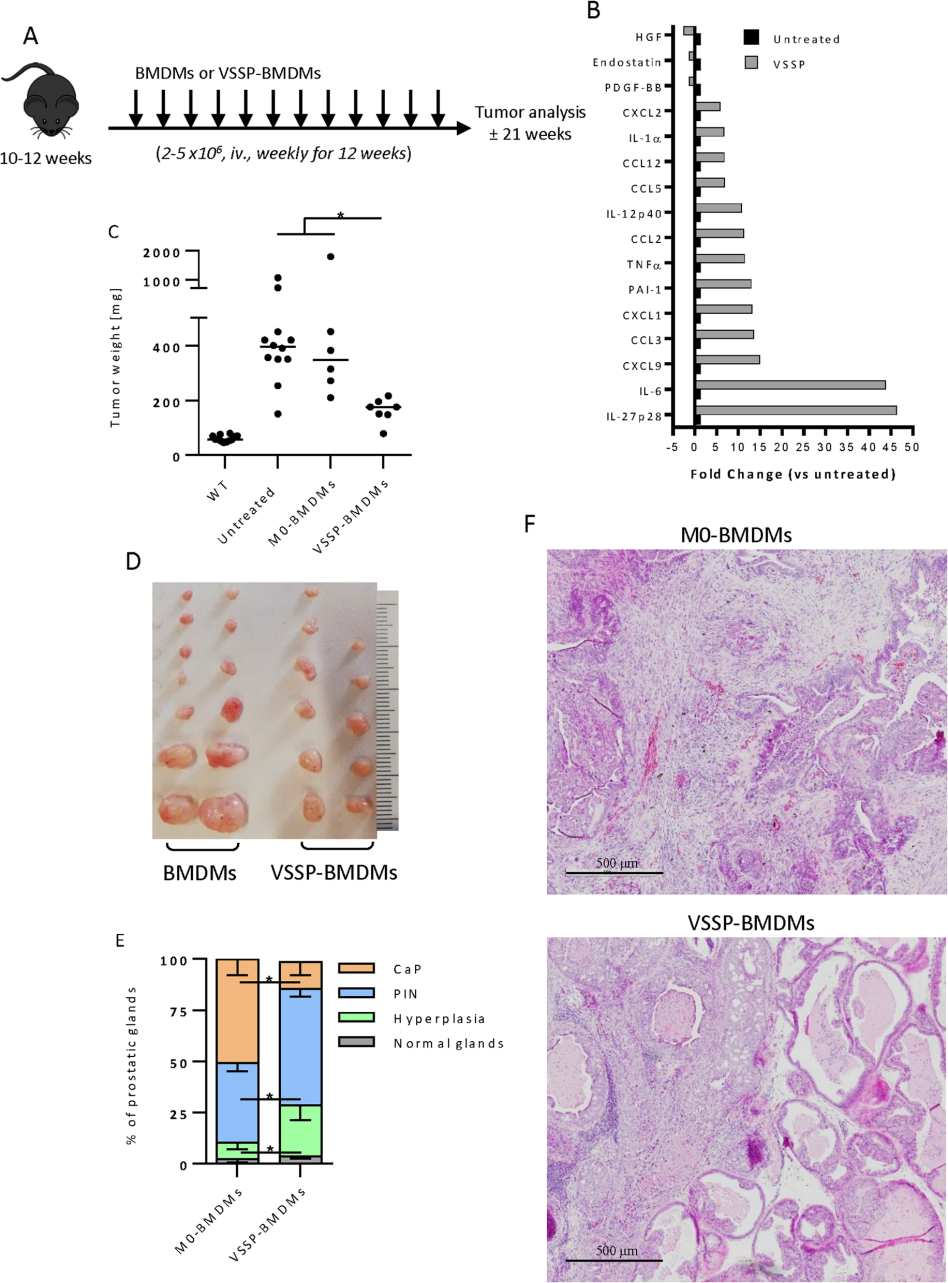
Adoptive transfer of VSSP-activated BMDMs reduce prostate tumor growth. **a** Schedule of treatment used in the pre-clinical trial with infused VSSP-activated BMDMs or steady state BMDMs on Pten^pc−/−^; Trp53^pc−/−^ tumor-bearing mice (n = 6–12 mice per group). 10–12 weeks old Pten^pc−/−^; Trp53^pc−/−^ tumor-bearing mice were infused weekly with 2–5 × 10^6^ BMDMs activated or not in vitro con VSSPs. After 12 weeks the mice were euthanized and tumors were collected. **b** Analyses of the conditioned medium of VSSP-BMDMs compared to untreated BMDMs, tested for the indicated soluble molecules by Proteome Profiler Mouse XL Cytokine Array. Bone marrow cells were cultured in RPMI with 10% heat-inactivated FBS in presence of 30 ng/mL of M-CSF. At day 5 media was replaced by RPMI containing 10 µg/mL of VSSP. Cells were cultured for 24 h and after, media was replaced by RPMI with 10% heat-inactivated FBS, and cells were cultured for 24 h to collect the CM. The graph shows the fold change of the indicated soluble molecules. Data is a metanalysis of three independent experiments. **c** Prostate weight compared with untreated Pten^pc−/−^; Trp53^pc−/−^ and wild type mice. **d** Representative picture of prostate lobes in Pten^pc−/−^; Trp53^pc−/−^ mice upon infusion of steady state or VSSP-activated BMDMs. **e** Quantification of adenocarcinoma, prostatic intraepithelial neoplasia (PIN), hyperplasia or normal-like glands in Pten^pc−/−^; Trp53^pc−/−^ mice upon infusion of steady state or VSSP-activated BMDMs. **f** Representative H&E at the endpoint. Scale Bar 500 μm. Data is representative of at least three biological independent animals. Data in **c** and **e** is a metanalysis of two independent experiments. Symbols indicates significant differences by Tukey test **c** or Student t test **e** (*p < 0.05)*.* *BMDMs* Bone marrow-derived macrophages

Since VSSP in vivo did not show antitumor effect in the Pten^pc−/−^; Trp53^pc−/−^ model, we wonder whether the administration of macrophages activated ex vivo with VSSP could inhibit Pten^pc−/−^; Trp53^pc−/−^ tumor growth. To this aim, we adoptively transfer BMDMs after in vitro stimulation with VSSP (VSSP-BMDMs) or their unstimulated counterparts into Pten^pc−/−^; Trp53^pc−/−^ mice, as shown in Fig. 2a. After in vitro stimulation with VSSP and prior to the infusion, we analyzed the macrophage phenotype by multiparametric flow cytometry. In line with previous unpublished results from our group, VSSP-BMDMs showed increased secretion of IL-12, TNFα and other M1-related proinflammatory cytokines compared to unstimulated BMDMs (Fig. 2b). Aligned with previous results of our group, VSSP-BMDMs showed a proinflammatory phenotype marked by the expression of classical M1 markers (CD40, CD80 and CD86) while reduced expression of M2 markers (CD206 and M-CSFR) (Alvarez-Arzola et al. manuscript in revision), as shown in Additional file 2: Fig. S2a. Previous studies demonstrated that CXCL2 chemokine is the major secreted factor from Pten^pc−/−^; Trp53^pc−/−^ tumor linked to M2 polarization of macrophages both in vitro and in the TME [3]. In order to evaluate if CXCL2 could interfere with the M1 polarization induced by VSSP on macrophages, we evaluated by FACS the expression of M1 markers on BMDMs upon in vitro stimulation with the combination of VSSP and CXCL2. Interestingly, VSSP increased M1 markers on BMDMs even in the presence of CXCL2 (Additional file 2: Fig. S2a). Summarizing, these results indicated that VSSP-BMDMs exhibit M1 phenotype and this property is not compromised by the presence of the M2-polarising factor CXCL2. We next evaluated the antitumor effect of VSSP-BMDMs adoptive transfer. After 12 weeks of treatment, VSSP-BMDMs significantly reduced the tumor burden in terms of tumor weight and tumor volume (Fig. 2c, d and Additional file 2: Fig. S2b). No change in the body weight was observed in the Pten^pc−/−^; Trp53^pc−/−^ mice receiving BMDMs adoptive transfer (Additional file 2: Fig. S2c). We also analyzed the tissue architecture and classified the prostatic glands according to the morphology and degree of malignant transformation. Histopathological analysis revealed a reduction in the tumor aggressiveness in VSSP-BMDMs infused mice, evidenced by a decreased number of glands with a phenotype of invasive carcinoma (Fig. 2e, f). So far, adoptive transfer of VSSP-BMDMs to Pten^pc−/−^; Trp53^pc−/−^ mice reduced tumor growth and aggressiveness.

### VSSP-BMDMs adoptive transfer activates CD8+ tumor-infiltrating T cells, reduces angiogenesis and induces senescence in Pten^pc−/−^; Trp53^pc−/−^ prostatic tumors

**Fig. 3:**
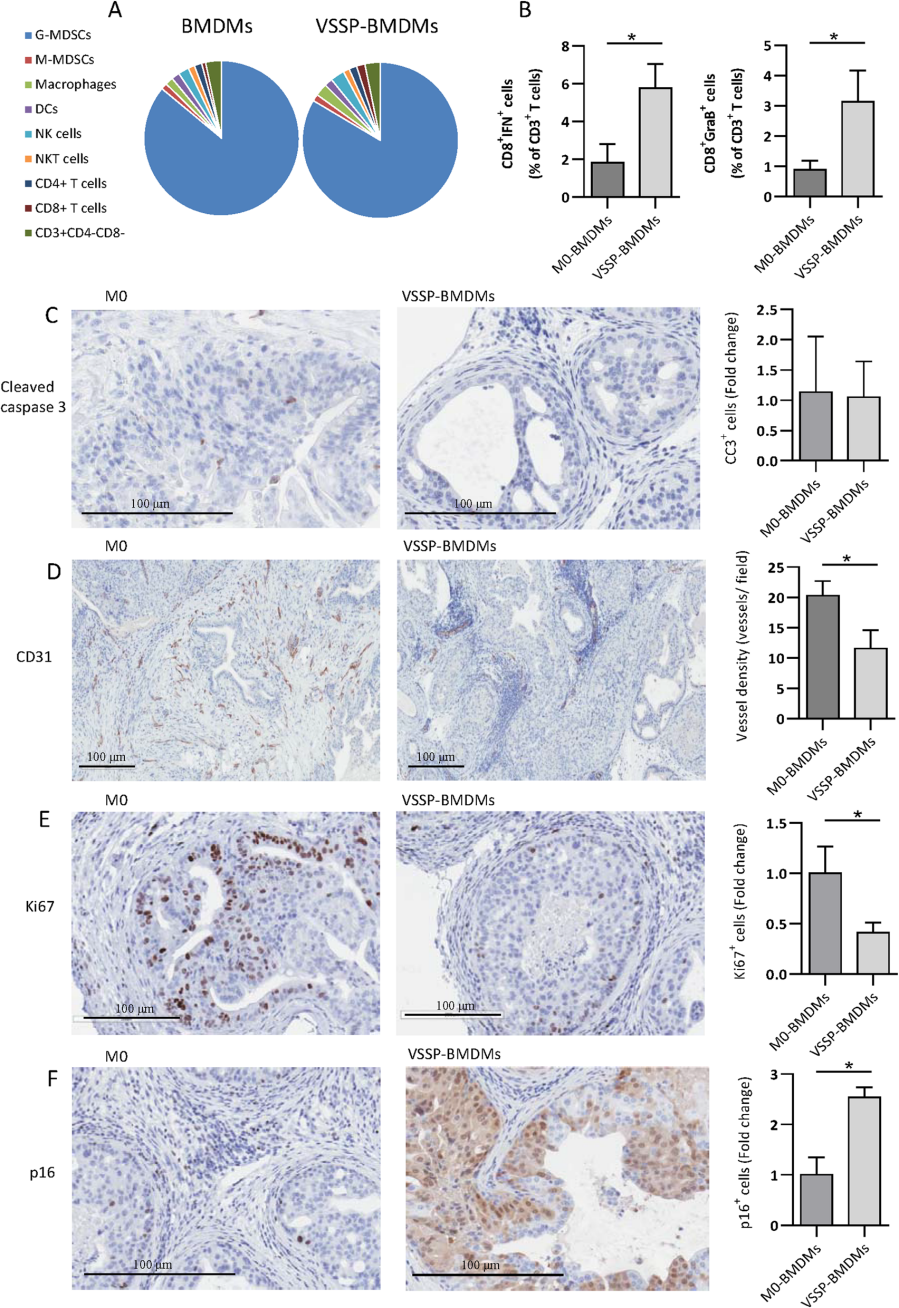
VSSP-activated BMDMs decrease angiogenesis, reduce proliferation and induce senescence in PCa cells in *vivo.* Steady state or VSSP-activated BMDMs were infused into Pten^pc−/−^; Trp53^pc−/−^ mice as described in Fig. 2a. **a** FACS quantitation of the immune cells infiltrating prostate tumor of Pten^pc−/−^; Trp53^pc−/−^ mice. Proportion of each immune population was estimated inside the CD45^+^ population. **b** FACS quantitation of the frequency of IFNγ^+^ and Granzyme B^+^ CD8^+^T cells in the prostate TME of mice infused with VSSP-stimulated and unstimulated BMDMs. **c** Representative IHQ staining of cleaved caspase-3 in the tumors at completion of the study. Scale Bar 100 μm. Quantification was performed as fold change of the percentage of positive cells within the glands. **d** Representative IHQ staining of CD31 in the tumors. Scale Bar 100 μm. Quantification was performed as density of vessels per field. **e** Representative IHQ staining of Ki-67 in the tumors. Scale Bar 200 μm. Quantification was performed as fold change of the percentage of positive cells within the glands. **f** Representative IHQ staining p16 in the tumors. Scale Bar 200 μm. Quantification was performed as fold change of the percentage of positive cells within the glands. Symbols indicates significant differences by Student t test (*p < 0.05)*.* *BMDMs* Bone marrow-derived macrophages, *MDSCs* myeloid-derived suppressor cells

To further investigate the mechanism by which VSSP-BMDMs control Pten^pc−/−^; Trp53^pc−/−^ tumor development, we evaluated the effect of the macrophage adoptive transfer on the tumor-associated immune system and the tumor biology. First, we analyzed by FACS the composition of the immune cells and their functionality in the prostate TME. The gating strategy used for immunoprofiling was the same used in the Pten^pc−/−^ model and depicted in Additional file 1: Fig. S1c. The analysis of the immune infiltrate of prostate tumors followed macrophage adoptive transfer did not show any difference between the groups (Fig. 3a). However, we observed an increase in interferon gamma (IFNγ)- and GraB-producing CD8+ T cells in the TME of Pten^pc−/−^; Trp53^pc−/−^ tumors (Fig. 3b). In the same experimental setting, a trend to an increase in perforin-producing CD8+ T cells was observed in the mice infused with VSSP-BMDMs (Additional file 2: Fig. S2d). To evaluate if the antitumor effect observed with the adoptive transfer of VSSP-BMDMs involved induction of apoptosis, we evaluated by IHC the expression of the apoptosis marker cleaved caspase3. No expression of this marker was detected in the tumors from any of the groups (Fig. 3c).

Previous studies demonstrated that the infusion of macrophages with dampened potential to acquire M2 phenotype to Pten^pc−/−^; Trp53^pc−/−^ prostate tumor-bearing mice mediated tumor control through inhibition of angiogenesis and induction of tumor cell senescence in the TME [3]. To evaluate whether VSSP-BMDMs were able to mediate an antiangiogenic effect, we evaluated by IHC the amount and morphology of intratumor blood vessels after BMDMs adoptive transfer. Notably, mice infused with VSSP-BMDMs had less content of blood vessels in the TME than the mice infused with control BMDMs (Fig. 3d). In accordance, the area and perimeter of the vessels was also reduced in mice receiving VSSP-BMDMs transfer (Additional file 2: Fig. S2e).

Next, we evaluated the proliferative potential of the tumor cells by measuring the expression of Ki-67 in the prostatic tumor glands. Interestingly, the tumors from the VSSP-BMDMs infused mice exhibited a significant reduction in the proliferating Ki-67+ cells (Fig. 3e). On the contrary, a marked increased expression of senescence-associated marker p16 was observed in tumors from mice receiving VSSP-BMDMs (Fig. 3f). We also observed an increased expression of senescence-associated markers H2AX, pHP1-γ and PAI-1 (Additional file 3: Fig. S3). Taken together, these results suggest that VSSP-BMDMs control prostatic tumor growth by reducing tumor angiogenesis and induction of senescence.

**Fig. 4:**
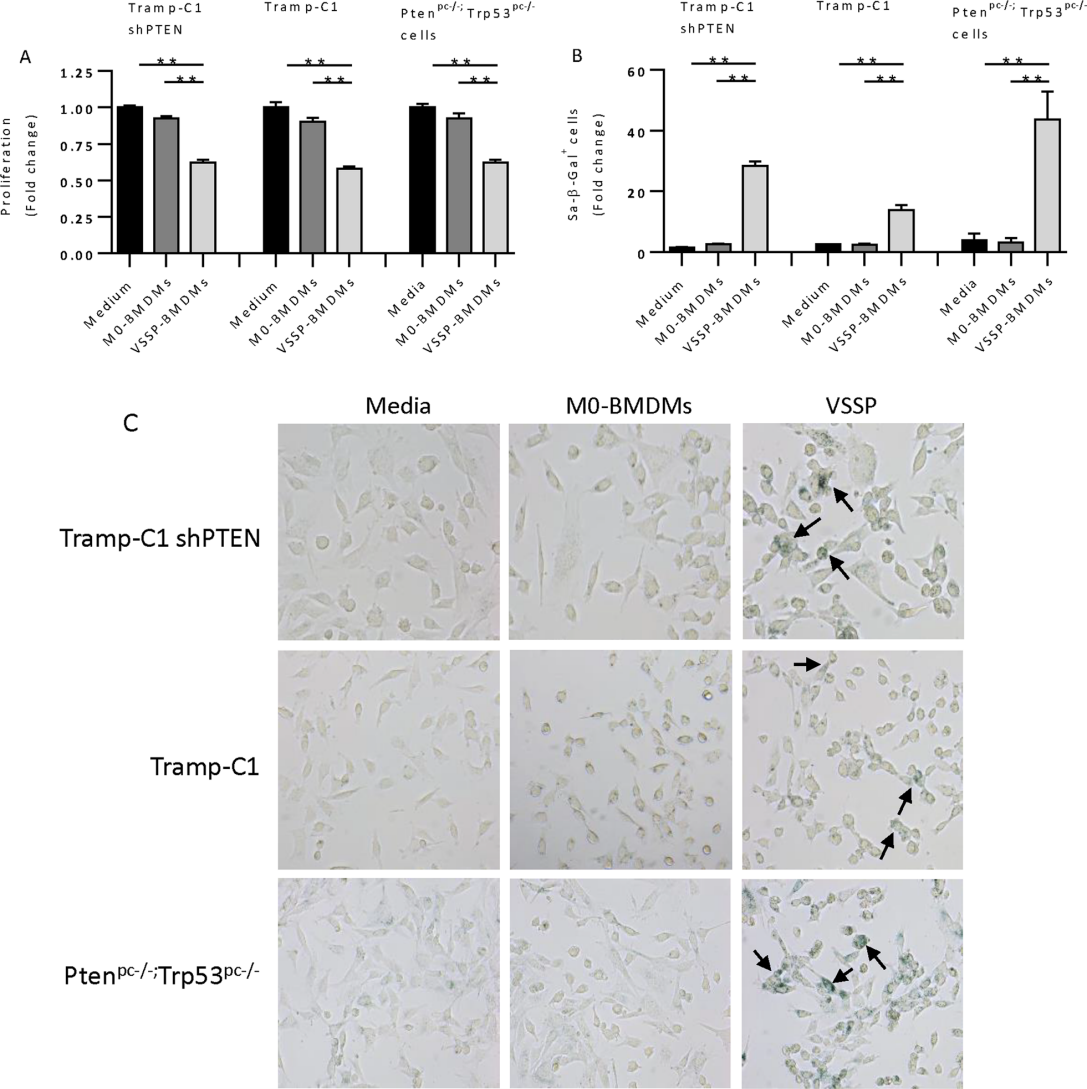
VSSP-activated BMDMs induce senescence in prostate cancer cells in vitro. PCa cell lines were cultured with RPMI 1640 containing 10% heat-inactivated FBS (normal medium), conditioned medium from BMDMs (1 part of normal medium and 1 part of unstimulated BMDMs conditioned medium) or conditioned medium from VSSP-activated BMDMs (1 part of normal medium and 1 part of VSSP-activated BMDMs CM). Then the cells were analyzed for Crystal Violet assay and/or for β-Galactosidase activity assay (after 72 h of culture, fold change compared with normal medium). **a** Proliferation assay on TrampC1-shPTEN cells, Tramp-C1 and Pten^pc−/−^;Trp53^pc−/−^ primary cells. Quantification was performed as fold change respect of the control. **b** Quantification of β-Galactosidase positive TrampC1-shPTEN, Tramp-C1 and Pten^pc−/−^;Trp53^pc−/−^ primary cells after treatment with the CM. Quantification was performed as fold change respect of the control. **c** Representative images of β-Galactosidase staining on TrampC1-shPTEN cells, Tramp-C1 and Pten^pc−/−^;Trp53^pc−/−^ primary cells. Positive stained cells are depicted by arrows. Symbols indicates significant differences by Student t test (**p < 0.01)*.* *BMDMs* bone marrow-derived macrophages

To better understand the mechanism linking VSSP-BMDMs and senescence induction, we cultured three isogenic PCa cell lines in the presence of the CM from BMDMs with and without VSSP stimulation. We used TRAMP-C1, TRAMP-C1-shPTEN and Pten^pc−/−^; Trp53^pc−/−^ primary cancer cell lines. The CM from VSSP-BMDMs significantly reduced the proliferation of all the cell lines compared to the CM derived from the unstimulated BMDMs (Fig. 4a). In addition, all cell lines cultured with CM from VSSP-BMDMs exhibited a significant increase in β-galactosidase activity compared to cells cultured in normal media (Fig. 4b, c). Collectively, these results confirmed that VSSP-BMDMs induce senescence in PCa cells.

## Discussion

Altogether our findings reinforce the notion that functional education of macrophages could impact CRPC therapeutic. By using an immunomodulator called VSSP, we demonstrated that the reduction of TAMs and the prevalence of M1 macrophages on the TME of murine prostate tumors inhibits tumor growth. In particular, we observed an antitumor effect in Pten^pc−/−^ tumor-bearing mice mediated by in vivo VSSP administration. In this model VSSP diminished TAMs content and promoted an M1 phenotype in the remaining TAMs. Additionally, in a more aggressive model (Pten^pc−/−^; Trp53^pc−/−^) that resembles CRPC in humans, the adoptive transfer of BMDMs previously polarized ex vivo to M1 using VSSP, induced tumor cell senescence and reduced tumor growth.

Cell-based therapies have arisen as a promissory strategy for some malignancies. In particular, adoptive cell therapy with genetically modified T cells has generated promising outcomes in hematological cancers; however, its efficacy in solid tumors faces many challenges [25]. There are several potential causes of the poor responses achieved by T-cell based therapies. Among other causes, effector cells have to migrate and penetrate the tumor through dense cellular and stromal barriers; circumvent hypoxia, acidic conditions and a diverse repertoire of suppressive cells that characterize TME [25–27]. Although novel strategies are under investigation to overcome some of these challenges (including expansion to other lymphocytes such as γδ T cells, natural killer T cells, and natural killer cells) [25], they still have to demonstrate their efficacy in the treatment of solid tumors and highlight the relevance of looking beyond lymphocyte-derived cells for potentially more relevant outcomes.

Macrophages are highly plastic cells with many functions, including regulation of tissue development and homeostasis, clearance of cellular debris, elimination of pathogens, and regulation of inflammatory responses in several contexts, including cancer [25, 28]. Tumors are characterized by the accumulation of macrophages in the TME. In facts, macrophages are the main immune population in the vast majority of tumor types. Precisely for the inherent ability of macrophages to penetrate both the dense stromal tissue surrounding tumors and the TME itself; their functional plasticity and the combination of direct tumoricidal activity with the potential to boost immunity through antigen presentation, highlight macrophages as a promising tool for antitumor cell therapy development [25, 28].

We demonstrated in this work that VSSP, a nanoparticulated modulator of the myeloid immune system, decreased TAMs levels in the TME of Pten^pc−/−^ prostate tumor-bearing mice. This result is consistent with recent evidences of Khan and colleagues who demonstrated that VSSP administration to ovarian tumor-bearing mice decreased TAMs levels on the peritoneal TME. In addition, the remaining TAMs exhibited an M1 phenotype with enhanced ability to induce T cell activation [29]. Interestingly, in our experiments in the Pten^pc−/−^ model, VSSP also induced an M1 activation pattern in the remaining TAMs, characterized by increased expression of CD80 and enhanced production of IL-12 and TNFα compared to the untreated counterparts. This effect on TAMs is associated with a significant inhibition of tumor growth. Despite the results obtained in the present study are similar to the ones described above in terms of the effect on TAMs, it is important to notice that the TME of the ovarian tumors is mainly peritoneal, which does not offer physical barriers for drugs administered directly to the peritoneum. PCa, like other solid tumors are less accessible and therefore, in many cases, the effect in the TME is compromised, especially for cell-based therapies. Accordingly, the evidences presented here constitute the first evidence of the efficacy of VSSP as a modulator of the TME in a solid tumor.

Previous work from our group have evidenced the systemic effect of VSSP. It has been documented that VSSP administration to healthy and tumor-bearing mice increased the splenic CD11b+Gr1+ myeloid cells with attenuated suppressive function [16, 17]. We also demonstrated recently that VSSP affects emergency myelopoiesis, shunting granulocyte-monocyte progenitor differentiation toward mononuclear phagocytes (monocytes, macrophages and DCs) in detriment of granulocytes [30]. Such evidences supported the role of VSSP as a systemic regulator of the myeloid immune system. However, the effect of VSSP on the TME was not fully characterized. In this work we centered our attention in the TME of prostatic tumors, specifically on macrophages, with the hypothesis that interfering with macrophage biology on the TME in addition to the effects that VSSP may have systemically, could be a better approach to target myeloid system in cancer. However, future studies should be performed to validate the possible systemic effects of long-lasting administration of VSSP in the particular setting of prostate cancer.

Even though VSSP was not effective as immunotherapy on the highly aggressive Pten^pc−/−^; Trp53^pc−/−^ model, the results in the Pten^pc−/−^ model validate the hypothesis of TAMs re-education as a promising therapy for PCa. Previous studies also validated this approach. Previous studies in the Pten^pc−/−^; Trp53^pc−/−^ tumor model have shown that the education of TAMs is mainly governed by the chemokine CXCL2, which mediates the M2 polarization. Therefore, treatment of Pten^pc−/−^; Trp53^pc−/−^ tumor-bearing mice with a CXCR2 inhibitor promotes TAMs re-education, leading to tumor inhibition [3]. There are current clinical trials in PCa patients using different monotherapies or therapies combined with ADT to induce TAMs re-education such as CSF-1R inhibitor (JNJ-40346527) (NCT03177460) [31], CXCR2 inhibitor (AZD5069) (NCT03177187) [24] and several trials using GM-CSF based combinations (NCT03600350, NCT03579654, NCT02961257, NCT03686683) [32–35]. However, no macrophage reprogramming therapy has been approved so far for its clinical use in PCa [36].

In addition to the therapies under investigation focused on TAMs re-education, a novel wave of therapies has arisen centered on the adoptive transfer of autologous ex vivo activated/modified macrophages [25, 28]. Actually, the use of chimeric antigen receptor macrophages is under phase I clinical trial evaluation in HER2 overexpressing solid tumors, including PCa (NCT04660929) [37]. In accordance with this rationale, we investigated the effectiveness of the adoptive transfer of BMDMs activated ex vivo with VSSP as an M1 polarizing agent. The administration of VSSP-BMDMs to Pten^pc−/−^; Trp53^pc−/−^ tumor-bearing mice significantly inhibited tumor growth compared to mice transferred with unstimulated macrophages. With the aim to evaluate if the phenotype of VSSP-BMDMs could be affected by the tumor-derived factors, we cultured BMDMs in the presence of VSSP and CXCL2, mimicking the influence of the Pten^pc−/−^; Trp53^pc−/−^ TME. Basically, VSSP induces an M1 phenotype (defined by increased co-stimulatory molecules and reduced expression of M2-related markers CD206 and M-CSFR), even in the presence of CXCL2. This result support the idea that VSSP-BMDMs should retain their M1 phenotype in the TME of the Pten^pc−/−^; Trp53^pc−/−^ tumors. However, additional experiments should be performed to validate this hypothesis.

Adoptive transfer of VSSP-BMDMs induced an antiangiogenic effect, evidenced by a reduction in density, area and perimeter in the blood vessels in the TME. Consistently with the M1 activation pattern demonstrated for the VSSP-BMDMs, the frequency of tumor-infiltrating CD8+ T cells secreting IFNγ and Granzyme B were increased in the TME, suggesting an immunostimulatory potential of VSSP-BMDMs. These results are aligned with previous evidences related to the ability of VSSP to determine and M1 polarization program on macrophages. Unpublished data from our group demonstrate that VSSP increases the expression of master regulators of the M1 polarization in steady state macrophages. Consequently, the phenotype, cytokine profile, and antigen-presenting cell features such as induction of T cell activation and proliferation, resembled canonical M1 macrophages (Alvarez-Arzola et al. manuscript in revision). Although the link between VSSP-activated macrophages and the CD8+ T cell functionality has been described in such scenarios, further studies should be performed to elucidate whether the effect on the CD8+ T cells in the Pten^pc−/−^; Trp53^pc−/−^ TME is mediated directly or indirectly by transferred VSSP-BMDMs.

Interestingly, IHC staining on tumor sections evidenced a reduction of the proliferation marker Ki-67 and increased expression of senescence-associated markers p16, H2AX, pHP1-γ and PAI-1 on mice infused with VSSP-BMDMs. The prosenescent properties of VSSP-activated BMDMs were also validated in vitro, since the CM from these macrophages reduced the proliferation and increased the activity of β-Galactosidase in three PCa cell lines. Di Mitri and colleagues previously described similar effects after infusion of CXCR2-deficient BMDMs to Pten^pc−/−^; Trp53^pc−/−^ tumor-bearing mice. Interestingly, the authors demonstrated that the induction of senescence was triggered by TNFα secreted by CXCR2-deficient macrophages [3]. From a mechanistic point of view, VSSP could be using a similar strategy to the above-mentioned approach since TNFα expression was increased in TAMs after in vivo administration of VSSP and in BMDMs following in vitro activation. It is important to notice that our results are similar in terms of efficacy compared to the previous preclinical studies referred in the same experimental setting. However, from a translational point of view, our strategy of using ex vivo M1-activated macrophages as effectors for adoptive therapy avoids the practical and technical issues associated with the genetic engineering process of other strategies. Taken together, we demonstrated that the adoptive transfer of BMDMs activated ex vivo with VSSP as an M1-polarizing agent, induced prostate tumor inhibition by increasing the functionality of tumor-infiltrating CD8+ T cells, reducing the density and the dimensions of the tumor-associated blood vessels and inducing tumor cell senescence.

Several scenarios arise from the present work that should be taken into consideration in further studies. Even when the adoptive transfer of VSSP-BMDMs was effective inhibiting tumor growth, combined therapies could be explored in order to potentiate the antitumor effect. Considering that adoptive transfer of VSSP-BMDMs induced tumor cell senescence, this therapy could be combined with senolytic agents such as inhibitors of BCL-2, BCL-XL and BCL-W and cyclin D-CDK4/6 complex inhibitor palbociclib, currently in clinical trials in several cancer types in combination with other therapies [30, 38–40]. In addition, the ability of VSSP-BMDMs to increase CD8+ T cell functionality in the TME could be a rationale for combining this therapy with CAR-T cell-based therapy in prostate cancer.

The results presented in this work support the rationale for further investigations centered on the use of macrophage reprogramming therapies as well as macrophage-based cell therapies, particularly ex vivo-activated macrophage adoptive transfer, as promissory therapies for PCa, specially CRPC.

## Supplementary Information

**Additional file 1: Fig. S1.** VSSP reduces Pten^pc−/−^ tumor weight and TAMs content and increases CD8+ *T cell functionality.* **a** 9–12 weeks old mice were surgically castrated and 7 days after started the administration of VSSP (5 mg/kg, i.p, twice per week), αCXCR2 (100 mg/kg, orally, daily) or vehicle (PBS, 200 µL; i.p. or orally). After 12 weeks of treatment mice were euthanized, tumors were collected and measured the tumor weight. **b** Pten^pc−/−^ mice body weight was measured weekly, starting with the first administration of treatments until the completion of the study. **c** Gating strategy for the profiling of the immune cells in the TME. For defining the cell populations, we excluded the singlets and gated the CD45^+^ cells inside the leukocyte region defined by FSC and SSC. Inside the CD45^+^ cells, M-MDSCs: CD11b^+^Ly6G^−^Ly6C^+^; G-MDSCs: CD45^+^CD11b^+^Ly6G^bright^Ly6C^low^; DCs: CD11b^+^Ly6G^−^Ly6C^−^F4/80^−^CD11c^+^; TAMs: CD11b^+^Ly6G^−^Ly6C^−^F4/80^+^; B cells: CD3^−^B220^+^; NK cells: CD3^−^NK1.1^+^: NKT cells: CD3^+^NK1.1^+^; CD4+ T cells: CD3^+^NK1.1^−^CD8^−^CD4^+^ and CD8+ T cells: CD3^+^NK1.1^−^CD4^−^CD8^+^. **d** Representative IHQ staining of F4/80 in the tumors at completion of the study. Scale Bar 100 μm. **e** FACS analysis of the expression of CD80, IL-12 and TNFα on tumor-infiltrating G-MDSCs from Pten^pc−/−^mice treated or not with VSSP. **f** FACS analysis of the expression of IFNγ and GraB on CD8+ TILs from Pten^pc−/−^ mice treated or not with VSSP. Symbols indicate significant differences by Tukey test (*p < 0.05). *G-MDSCs* granulocytic myeloid-derived suppressor cells, *CTX* surgical castration, *TILs* tumor-infiltrating lymphocytes.

**Additional file 2: Fig. S2.** VSSP-BMDMs exhibit an M1 phenotype and reduces tumor volume and angiogenesis when transferred to Pten^pc−/−^; Trp53^pc−/−^. **a** FACS analysis of the BMDMs phenotype after in vitro stimulation. Bone marrow cells were cultured in RPMI with 10% heat-inactivated FBS in presence of 30 ng/mL of M-CSF. At day 5 media was replaced by RPMI containing with VSSP (10 µg/mL), IL-4 (30 ng/mL) + IL-13 (30 ng/mL), CXCL2 (100 ng/mL), CXCL2 + VSSP, IFN (10 ng/mL) or media alone and cells were cultured for 24 h. **b** 10–12 weeks old Pten^pc−/−^; Trp53^pc−/−^ tumor-bearing mice were infused weekly with 2–5 × 10^6^ BMDMs activated or not in vitro con VSSPs. After 12 weeks the mice were euthanized, tumors were collected and measured the tumor volume. **c** Pten^pc−/−^; Trp53^pc−/−^mice body weight was measured weekly, starting with the first adoptive transfer until the completion of the study. **d** FACS quantitation of the frequency of Perforin + CD8+ T cells in the prostate TME of mice infused with VSSP-stimulated and unstimulated BMDMs. **e** Quantification of blood vessel area and perimeter in the same experimental setting described in **b**. Symbols indicate significant differences by ANOVA in **b** and Student t test in **e** (*p < 0.05; **p < 0.01). *BMDMs* Bone marrow-derived macrophages.

**Additional file 3: Fig. S3.** Adoptive transfer of VSSP-activated BMDMs induce senescence in Pten^pc−/−^; Trp53^pc−/−^ tumors. Steady state or VSSP-activated BMDMs were infused into Pten^pc−/−^; Trp53^pc−/−^ mice as described in Fig. 2a. Representative IHQ staining of PHP1γ **a**, H2AX **b** and PAI-1 **c** in the tumors at completion of the study. Scale Bar 100 μm. *BMDMs* Bone marrow-derived macrophages.

## Data Availability

All data generated and analyzed during this study are included in this article and its supplementary material files. Further enquiries can be directed to the corresponding author.
