# Supplementary figures and images for: VSSP-activated macrophages mediate senescence and tumor inhibition in a preclinical model of advanced prostate cancer
Source: Cell Commun Signal. 2023 Apr 13;21:76. doi: 10.1186/s12964-023-01095-3 (PMC10100133; doi:10.1186/s12964-023-01095-3)

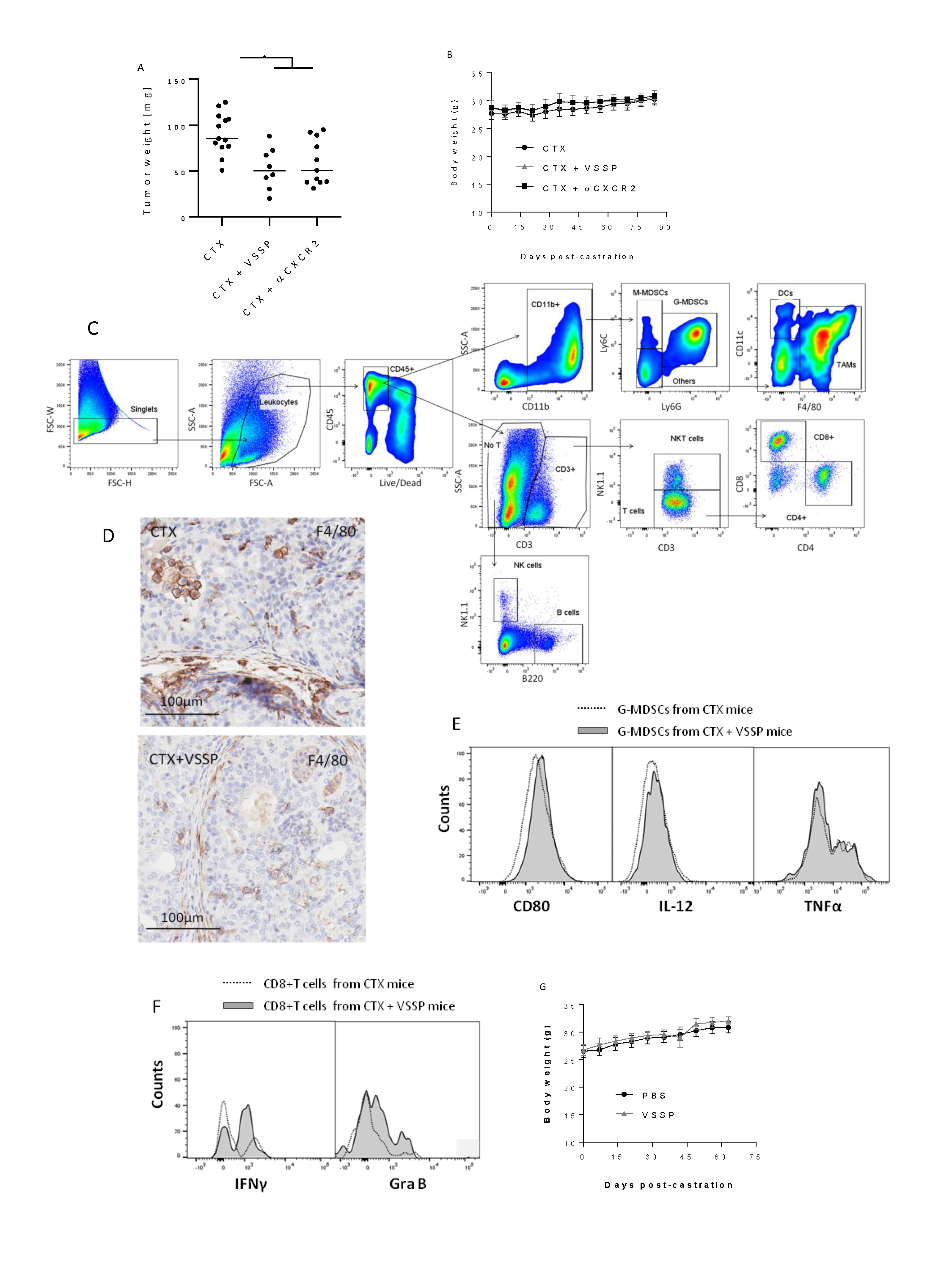

Supplement: Supplementary file 2 — Additional file 1: Fig. S1. VSSP reduces Ptenpc−/− tumor weight and TAMs content and increases CD8+ T cell functionality. a 9–12 weeks old mice were surgically castrated and 7 days after started the administration of VSSP (5 mg/kg, i.p, twice per week), αCXCR2 (100 mg/kg, orally, daily) or vehicle (PBS, 200 µL; i.p. or orally). After 12 weeks of treatment mice were euthanized, tumors were collected and measured the tumor weight. b Ptenpc−/− mice body weight was measured weekly, starting with the first administration of treatments until the completion of the study. c Gating strategy for the profiling of the immune cells in the TME. For defining the cell populations, we excluded the singlets and gated the CD45+ cells inside the leukocyte region defined by FSC and SSC. Inside the CD45+ cells, M-MDSCs: CD11b+Ly6G−Ly6C+; G-MDSCs: CD45+CD11b+Ly6GbrightLy6Clow; DCs: CD11b+Ly6G−Ly6C−F4/80−CD11c+; TAMs: CD11b+Ly6G−Ly6C−F4/80+; B cells: CD3−B220+; NK cells: CD3−NK1.1+: NKT cells: CD3+NK1.1+; CD4+ T cells: CD3+NK1.1−CD8−CD4+ and CD8+ T cells: CD3+NK1.1−CD4−CD8+. d Representative IHQ staining of F4/80 in the tumors at completion of the study. Scale Bar 100 μm. e FACS analysis of the expression of CD80, IL-12 and TNFα on tumor-infiltrating G-MDSCs from Ptenpc−/−mice treated or not with VSSP. f FACS analysis of the expression of IFNγ and GraB on CD8+ TILs from Ptenpc−/− mice treated or not with VSSP. Symbols indicate significant differences by Tukey test (*p < 0.05). G-MDSCs granulocytic myeloid-derived suppressor cells, CTX surgical castration, TILs tumor-infiltrating lymphocytes. [file 12964_2023_1095_MOESM2_ESM.tif]

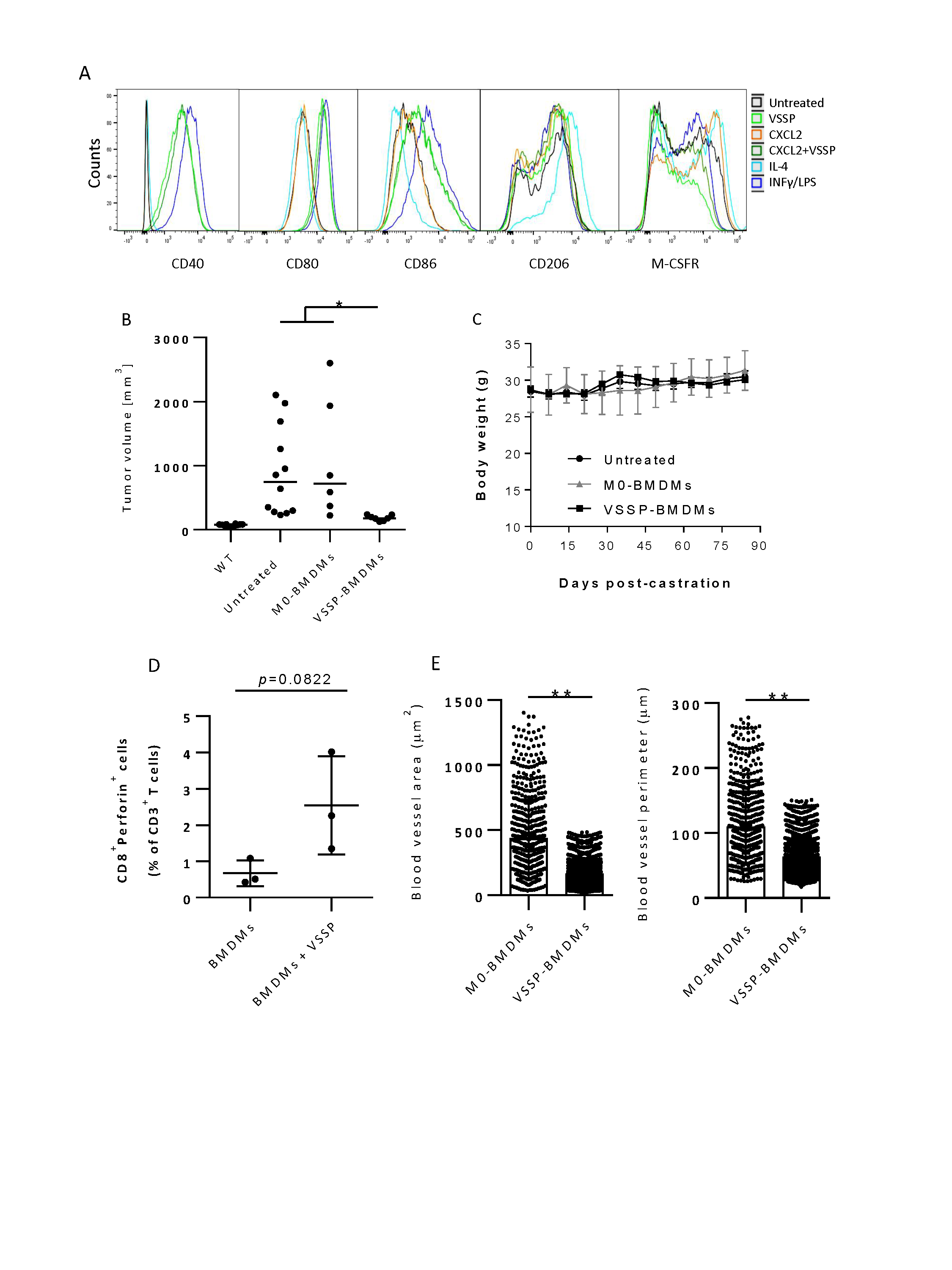

Supplement: Supplementary file 3 — Additional file 2: Fig. S2. VSSP-BMDMs exhibit an M1 phenotype and reduces tumor volume and angiogenesis when transferred to Ptenpc−/−; Trp53pc−/−. a FACS analysis of the BMDMs phenotype after in vitro stimulation. Bone marrow cells were cultured in RPMI with 10% heat-inactivated FBS in presence of 30 ng/mL of M-CSF. At day 5 media was replaced by RPMI containing with VSSP (10 µg/mL), IL-4 (30 ng/mL) + IL-13 (30 ng/mL), CXCL2 (100 ng/mL), CXCL2 + VSSP, IFN (10 ng/mL) or media alone and cells were cultured for 24 h. b 10–12 weeks old Ptenpc−/−; Trp53pc−/− tumor-bearing mice were infused weekly with 2–5 × 10^6^ BMDMs activated or not in vitro con VSSPs. After 12 weeks the mice were euthanized, tumors were collected and measured the tumor volume. c Ptenpc−/−; Trp53pc−/−mice body weight was measured weekly, starting with the first adoptive transfer until the completion of the study. d FACS quantitation of the frequency of Perforin + CD8+ T cells in the prostate TME of mice infused with VSSP-stimulated and unstimulated BMDMs. e Quantification of blood vessel area and perimeter in the same experimental setting described in b. Symbols indicate significant differences by ANOVA in b and Student t test in e (*p < 0.05; **p < 0.01). BMDMs Bone marrow-derived macrophages. [file 12964_2023_1095_MOESM3_ESM.tif]

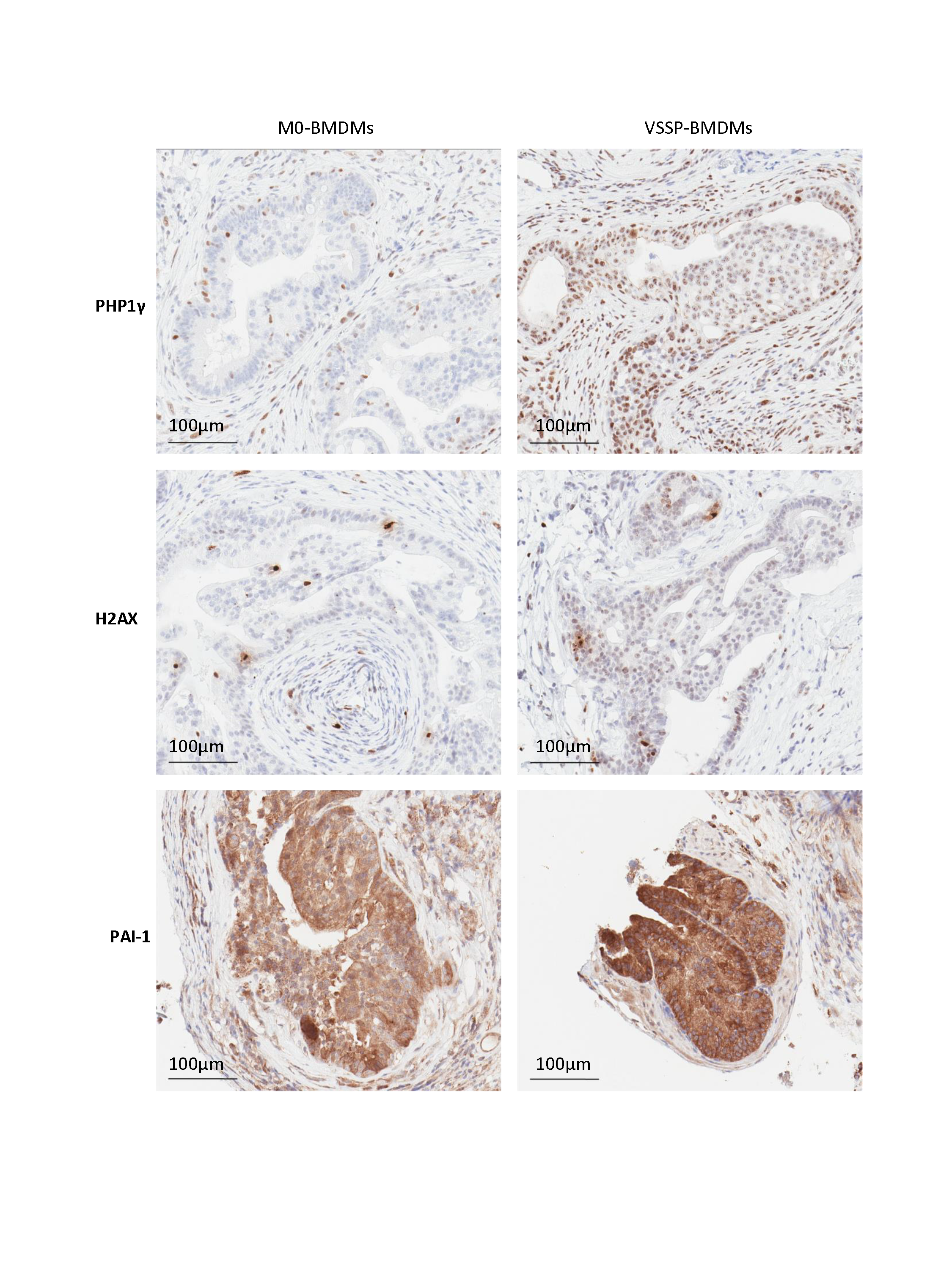

Supplement: Supplementary file 4 — Additional file 3: Fig. S3. Adoptive transfer of VSSP-activated BMDMs induce senescence in Ptenpc−/−; Trp53pc−/− tumors. Steady state or VSSP-activated BMDMs were infused into Ptenpc−/−; Trp53pc−/− mice as described in Fig. 2a. Representative IHQ staining of PHP1γ a, H2AX b and PAI-1 c in the tumors at completion of the study. Scale Bar 100 μm. BMDMs Bone marrow-derived macrophages. [file 12964_2023_1095_MOESM4_ESM.tif]
